# Classification in Para skiing: do better performing skiers have better visual functions?

**DOI:** 10.3389/fspor.2023.1046318

**Published:** 2023-04-17

**Authors:** Amritha Stalin, Kristine Dalton

**Affiliations:** School of Optometry & Vision Science, University of Waterloo, Waterloo, ON, Canada

**Keywords:** para nordic skiing, para alpine skiing, sports classes, visual acuity, visual field

## Abstract

**Introduction:**

Currently, Paralympic skiers with vision impairment are allocated to classes based only on their better eye static visual acuity and visual field diameter. These studies were conducted to investigate whether a broad range of visual functions were different among groups of skiers with different levels of skiing performance.

**Methods:**

Static and dynamic visual acuities, contrast sensitivity, light and glare sensitivity, glare recovery, motion perception, and visual field were assessed binocularly in elite Para nordic (*n* = 26) and Para alpine skiers (*n* = 15) at 3 international Paralympic events. Skiing performances were calculated using modified skiing points systems based on skiers' raw race times. Clusters of skiers with similar performances were identified in each sport, and their vision and non-vision variables were compared.

**Results:**

Skiers in the best performing Para nordic clusters (1 and 2) had better static visual acuities (*p* = 0.041) and larger visual fields (*p* = 0.004) compared to cluster 3. In Para alpine slalom (*p* = 0.019), giant slalom (*p* = 0.019), and Super-G (*p* = 0.039) the average static visual acuities among the better performing clusters were significantly better compared to the worst performing cluster. In slalom, the cluster with better performance also had a significantly larger visual field (*p* = 0.038). In downhill, the better performance cluster demonstrated better dynamic visual acuity (*p* = 0.029).

**Discussion:**

Clusters with better performing skiers appear to have better visual function in both sports. The results of this study would suggest that Para nordic and Para alpine skiers with light perception or no light perception vision should be in one class and that the skiers with quantifiable static VA should be in a different class.

## 1. Introduction

Paralympic classification systems encourage participation in sport by individuals with disabilities by reducing the effect of their impairments on sports performance outcomes (1). Through classification, the International Sport Federations allocate eligible athletes to classes or groups such that the impact of athletes' impairments on the sports performance is similar within each class. The current sports class allocation systems in the visually impaired (VI) category of Para nordic and Para alpine skiing are based only on two measures of visual function: static visual acuity (VA) and visual field (VF) of the better eye. Impairments in other visual functions such as contrast sensitivity (CS) or dynamic VA, which may impact skiing performance are not taken into consideration currently. Skiers with static VAs worse than 2.6 logMAR, including those with and without light perception vision are allocated into the B1 class. Skiers with static VAs ranging from 1.5 logMAR to <2.6 logMAR or a VF diameter of less than or equal to 10 degrees are classified as B2. The B3 class, with the least severe vision impairments, has skiers with static VAs ranging from 1.0 logMAR to <1.5 logMAR or a VF diameter of less than or equal to 40 degrees (2). During the events, skiers with VI in all classes compete together for one medal, and the finish time is determined to 1/100 precision (e.g., 3:22.38). A class factor is used to adjust the race times such that skiers with most severe impairments receive a maximum time bonus. These adjusted race times will be used to determine the overall results of the race (3, 4). The Para nordic skiing percentages are the same for all events (sprint, short distance, middle distance, and long-distance). The Para alpine skiing percentages assigned for B1, B2, and B3 in various disciplines are calculated separately for each discipline. In both sports, the percentages are determined by the Sports Technical Committees and are re-evaluated after every season if needed.

A four-round Delphi study completed in 2016 with 25 VI sports experts had concluded that current classification systems do not account for the unique visual demands or the performance environments of each sport and that new evidence-based systems should be designed (5). Based on the guidelines provided by the classification code (6) and joint position statement (7), various studies have been conducted by experts in different Para sports such as shooting (8, 9), swimming (10, 11), and Judo (12–14) to design sports-specific evidence-based classification systems (10).

Both Para nordic and Para alpine skiers have to make quick decisions about their speed, direction, and body position in response to visual information from the surrounding terrain and course markings (15). The vision demands involved in skiing suggest that visual functions such as VA, CS, and depth perception might be crucial for competitive skiing (16, 17). Since skiers often move at high speeds, it is also necessary to assess the significance of static and dynamic measures of these visual functions whenever possible. Although skiing is thought to depend more on central vision than peripheral vision, skiers still need to process information from larger visual spaces than most of the other sports like tennis or swimming, which suggests that there are also peripheral vision demands while skiing (16). One of the earliest studies conducted on alpine skiers in 1955 reported that the occlusion of peripheral vision caused marked deterioration in the motor control of skiers, compared to occlusion of central vision (18). Furthermore, skiers are exposed to glare from the snow and bright sunlight in their environment, as well as shadows cast by the trees. Also, skiers' visibility can be greatly altered by changes in the weather, lighting conditions, and the conditions of snow, especially while moving from sunny to shady parts of courses (16).

While nordic and alpine skiing are similar in terms of the high visual demands and the challenging environments, they differ in aspects related to the terrain and skiing techniques. Although both nordic and alpine skiing are physically demanding, Stöggl et. al reported that the energy spent by a skier during an hour of nordic skiing is equivalent to that spent during 2.5 h of alpine skiing (19). However, due to the speed involved, the risk of injuries are higher in Para alpine skiing, compared to Para nordic skiing (20). Nordic skiing is practiced on groomed trails on flatter and often tree-laden terrains, which are in loops, with uphills, gentle downhills, and curves (21). Groomed alpine courses are shorter, steeper and wider compared to the nordic courses and skiers race only downhill. There could be multiple skiers on the nordic course in some events, while on alpine courses there is typically only one skier (or one skier and their guide) competing at a time (22), therefore nordic skiers might need to utilize visual information from a wider field to avoid collision with other skiers or to look around trees or curves, compared to the alpine skiers. However, alpine skiers might need faster processing of visual information to make quicker decisions on adjusting their speed and positions, compared to nordic skiers. These unique demands of both sports suggests that the effects of VI on skiing performance needs to be explored separately in Para nordic and Para alpine skiing. Also, in Para nordic skiing, the use of guides is optional for athletes in B2 and B3 categories, whereas it is obligatory for athletes in the B1 class. All Para alpine skiers are required to use guides during competitions.

Within each of these sports, there are also different events or disciplines that athletes compete in. The three main individual competition events in Para nordic skiing are sprint, middle distance, and long distance, which differ only in terms of the length of the ski courses. In Para alpine skiing, there are four major disciplines, which differ in terms of course design as well as skiing technique. Downhill (DH) is a technical event in high-speed environment, where skiers race long, steep courses that include relatively few gates, turns, and jumps. Slalom (SL) is a technical event with shorter courses and a higher number of gates and turns compared to the other Para alpine disciplines. Giant slalom (GS) is also a technical event with longer courses and fewer wider and smoother turns than SL. Super G (SG) combines the speed of DH with the turns of GS and requires speed as well as technical skill.

Initial studies and observations conducted on elite Para nordic and Para alpine populations suggested that out of all vision measurements, static VA and VF were sufficient to explain the changes in the skiing performances once age and training factors were taken into account (23). However, the differences in the distributions of other vision and non-vision variables between higher performing and lower performing skiers were not explored in these studies. This project was designed to inform the development of sports class allocation criteria for Para nordic and Para alpine skiing by investigating various visual functions that could significantly affect skiing performance. This purpose of this study was to examine the existence of clusters, or groups of skiers with similar skiing performances, within currently classified elite Para nordic and Para alpine populations, and to compare the vision and non-vision related variables among these groups in each sport. The Para nordic competition events of different distances were examined as a pooled group because the three individual Para nordic competition events differ only in terms of the length of the course and are very similar in terms of the visual demands they require. The four Para alpine disciplines were examined separately as the visual demands might be significantly different among them due to the differences in terrain, gate settings, and skiing techniques involved. The test battery used to assess visual functions was based on previous research conducted by our group (24–26).

## 2. Materials and methods

The two independent studies conducted in Para nordic and Para alpine skiing used observational designs. Informed consent was obtained from all participants, and the studies adhered with the tenets of the Declaration of Helsinki. The studies were reviewed by and received ethics clearance through a University of Waterloo Research Ethics Committee.

An initial analysis of the data was conducted to identify the relationships between skiing performance and a wide range of visual functions in elite Para nordic and Para alpine skiers and the findings were published by Stalin et al. (23) This manuscript describes the second part of the data analysis, which explored these relationships further by evaluating these differences between skiers with significantly different skiing performances. The purpose of the data analysis described here, was to determine the sport class allocation for both sports. Elite Para nordic and Para alpine skiers were recruited with the help of the World Para Snow Sport at the 2017 Para Nordic World Championships (Finsterau, Germany), 2018 Para Nordic World Cup (Oberried, Germany), and the 2017 Para Alpine World Championships (Tarvisio, Italy). Binocular tests of static VA, dynamic VA, CS, glare sensitivity (GLS), glare recovery (GLR), light sensitivity (LS), translational motion perception (MP), radial MP, and VF were used to measure athletes' visual function. Skiing performance was assessed using recalculated, World Para Nordic Skiing (raw-WPNS) and World Para Alpine Skiing (raw-WPAS) points. Skiers' race points across the season were recalculated using raw race times without the class factor and used as the performance measure to ensure that the outcome measure was not impacted by skiers' previous classification. In par with the current sports rules, skiers' best five performances in a 24-month window were used to determine raw-WPNS points, while skiers' best two performances in a 15-month period were used for raw-WPAS points (3, 4). The performance points in WPAS are discipline specific (DH, SG, GS, and SL), but not in WPNS.

The formula for calculating unfactored race points was $P=\left(\left(\frac{Tx}{T0}\right)-1\right)*F+race\;pentalty$, where *P* = race points, T_X _= raw race time of competitor in seconds, T_0 _= raw race time of the overall gender best performer in seconds, and F = discipline factor. The race penalty is another factor determined by the IPC to account for the quality of competition and ensures that race points from different competitions can be compared equitably. Using this formula, best skiers have the lowest performance points (3, 4). This formula calculates race points relative to the race time of the overall best performer in each race, for each gender. As performance points were normalized to the best performance in each gender and visual function does not appear to differ between genders, researchers were able to compare performance data between genders, which was important because of the small number of elite alpine and nordic skiers with vision impairment in the world.

Further details regarding participant recruitment, the visual function assessments conducted, and the skiing performance points calculations can be found in our previous manuscript (23). The non-vision related performance variables accounted for in this analysis included age, age started skiing, age of onset of impairment, total lifetime hours of skiing, and number of races during the validity period. Data on non-vision related performance variables were collected using a standardized questionnaire that was completed by all athletes (see Stalin et al. for more details) (27).

### 2.1. Data analysis

Data analysis was conducted using SPSS for Windows (version 25.0, SPSS, Inc.) and SPSS modeler (version 18.2.1). The raw-WPNS and raw-WPAS points were not normally distributed, thus non-parametric tests were used for further analysis. Hierarchical cluster analysis was used to identify natural clusters of data within the Para nordic and Para alpine participants based on similarities in skiing performance. Vision and non-vision related variables were then compared between the different performance clusters were using the Kruskal-Wallis test with the Dunn-Bonferroni *post hoc* test when there were more than two clusters, or a Mann-Whitney *U post hoc* test when there were only two clusters. The Dunn-Bonferroni or Mann-Whitney *U post hoc* tests were carried out for each pair of groups to calculate the adjusted significance when Kruskal Wallis tests were found to be significant. According to the previously published results, both nordic and alpine participants' non-vision variables did not appear to be significantly predictive of skiing performance in nordic or alpine group and were generally uncorrelated with vision variables (23). However, non-vision variables were included in this analysis, because it allowed us to again compare the non-vision variables between the performance clusters and determine whether these variables were unexpectedly different between the groups (e.g., had the potential to overshadow vision variables).

An agglomerative hierarchical clustering algorithm was used to determine the clusters based on the performance points, which starts the process by considering each participant as a separate cluster. In each subsequent step, the two clusters that were most similar were combined to form a single cluster. This process continued until all clusters were combined finally to form one large cluster. For the analysis completed here, clusters were identified solely on the participants' skiing performance points, which means that all participants, even those who did not have measurable CS or DVA, were included in the analysis. Arbitrary values of 3.8 logMAR and 4.2 logMAR were assigned for the static VA of skiers with light perception (LP) and no light perception (NLP) vision, respectively to include their data in the correlation and regression analyses on the same continuous scale as the other participants. Similarly, zero (0.0 logCS or 0% VF) values were assigned for the CS and VF of participants with LP or NLP vision. Assigning the same arbitrary value of zero for these participants' dynamic VA, GLS, GLR, and LS would mean that they did not have a change in VA with the induced motion, glare, or light. Similarly, assigning 100% as arbitrary value for their translational MP & radial MP would mean that they were able to perceive motion at 100% coherence. Therefore, it was not possible to appropriately assign arbitrary values for dynamic VA, GLS, GLR, LS, translational MP, or radial MP for participants with LP or NLP vision. However, the missing or unmeasurable dynamic VA, GLS, GLR, LS, translational MP, or radial MP data did not affect the clusters formed because the clusters were formed based only on performance points, which all athletes had.

Ward's linkage criterion was used to determine the similarity of each cluster and identified the two most similar clusters by assessing the sum of squares of distances between the clusters. Ward's method is known to produce clusters with the same shape and roughly the same number of observations (28). The cluster solution obtained prior to a large change in the coefficient of distances between the clusters was chosen as the best solution. Hierarchical structure of the dendrograms were also checked to confirm the optimal clusters. The nearest neighbour criterion was used initially to identify potential outliers and the average linkage criterion was used to check the consistency of the results obtained through Ward's linkage (29). As the cluster results are known to occasionally vary depending on the distance measure used, the cluster analyses were repeated using the average linkage method to assess the consistency of the cluster results.

In addition to the cluster analysis, a decision tree analysis was used in the Para nordic data to identify the best predictor of skiing performance. Expected performance was defined as the average raw-WPNS points in cluster 1 (best performing cluster), and “below-expected” performance was defined as any performance points that exceeded the upper 99% confidence interval around the average raw-WPNS of cluster 1 (30). Decision trees differ from hierarchical clustering as decision trees are created to maximize the leaf purity of skiing performances. There is no such target for the cluster tree, which groups the clusters based on the similarity between each cluster. Both hierarchical clustering and the decision tree analysis were considered in determining the sports class criteria for Para nordic. Unfortunately, decision tree analysis was not possible for Para alpine due to fewer participants in the study and unequal cluster sizes generated by the hierarchical cluster analysis. Thus, the Para alpine sport class criteria were determined based on hierarchical clustering alone.

## 3. Results

A total of 26 Para nordic skiers (18 male, 8 female; age 26.0 ± 6.3 years, range: 18 to 43 years) from 13 nations and 15 Para alpine skiers (8 male, 7 female; age 28.1 ± 11.6 years, range 16 to 58 years) from 10 nations participated in these studies. Detailed participant demographics and a summary of the visual functions of participants per sport, can be found in the previous manuscript (23).

### 3.1. Skiing performance

The average raw-WPNS points of Para nordic participants was 58.73 ± 52.44 (range: 0.00 to 172.07, *N* = 26). The average raw-WPAS points of Para alpine participants for DH discipline was 155.81 ± 66.36 (range: 33.99 to 254.19, *N* = 9), GS was 226.98 ± 212.13 (range: 51.11 to 854.02, *N* = 15), SG was 336.20 ± 341.34 (range: 50.09 to 1299.41, *N* = 13), and SL was 193.40 ± 185.03 (range: 66.77 to 722.13, *N* = 15).

No outliers were identified in the Para nordic or Para alpine data sets based on the nearest neighbour criterion. Both the Ward's linkage and average linkage methods generated the same performance clusters, which suggests that the cluster results obtained through Ward's linkage method are valid. Therefore, none of the cluster conclusions would have changed if the average-linkage method had been used instead of the Ward's linkage criterion.

### 3.2. Para nordic skiing performance clusters

**Figure 1:**
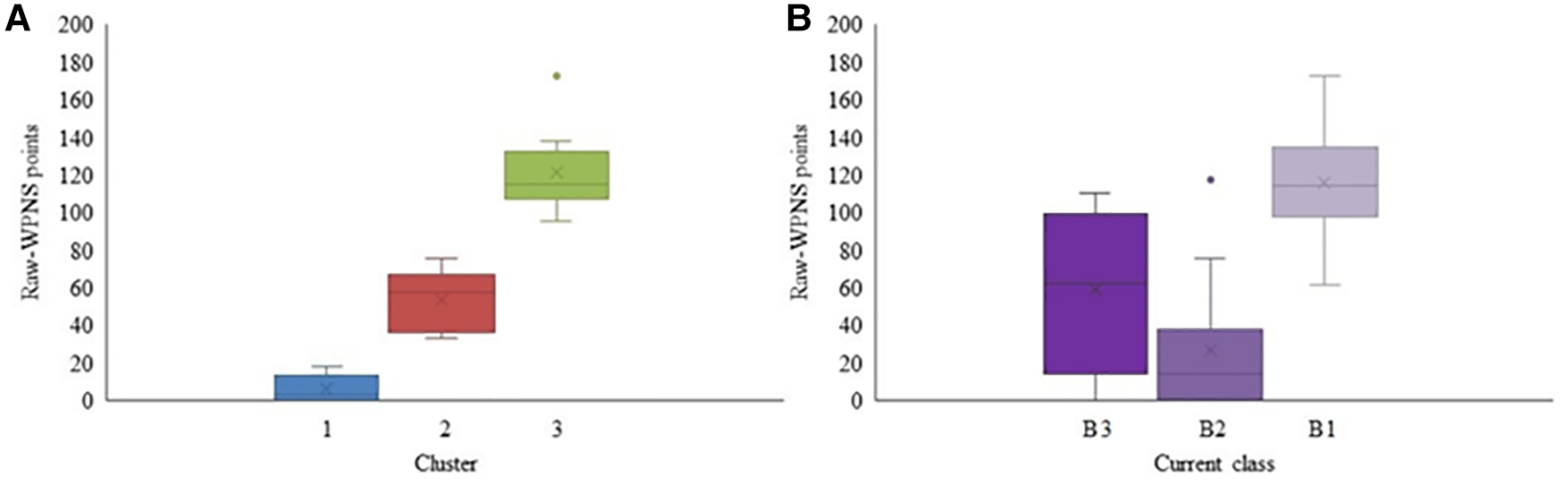
Raw-WPNS points for each cluster (**A**) and Raw-WPNS points for each current competition class (**B**) for the Para nordic participants. Mean value is marked with × in each box.

The cluster analysis in the Para nordic data resulted in three distinct clusters based on the WPNS points (*p* < 0.001; Figure 1A). Raw-WPNS points of participants based on their current classes are provided for comparison purposes in Figure 1B. There was an overall significant difference among the skiers' performance based on their current class (*p* = 0.002). Post hoc analyses suggested that the performances of B1 skiers were significantly different from that of B2 skiers (*p* = 0.001).

The summary statistics of the vision and non-vision variables in each cluster are shown in Table 1. In addition to the performance points, static VA (*p* = 0.041), VF (*p* = 0.004), and the number of races (*p* = 0.044) were significantly different among the clusters. The first cluster had participants with the best performance (lowest average performance points), better static VAs, and larger VF extents. Cluster 3 had participants with the worst average performance and cluster 2 had performance points in between clusters 1 and 3. The average static VA and VF between clusters 1 and 3 (adjusted significance values: Static VA *p* = 0.029; VF *p* = 0.002), and between 2 and 3 (adjusted significance values: Static VA *p* = 0.030; VF *p* = 0.010) were significantly different. The average number of races in cluster 3 was significantly different from cluster 1 (adjusted significance: *p* = 0.015), but clusters 2 and 3 were not significantly different (adjusted significance: *p* = 0.492). Cluster 1 and 2 were not significantly different in any vision or non-vision related aspects except in the raw-WPNS points.

**Table 1:** Summary statistics (mean ± SD, median) of vision and non-vision related variables the Para nordic clusters. The values of variables that are significantly different among the clusters are bolded. The values of non-vision variables are italicized. The summary data include only actual measured values and not the arbitrarily assigned values of VA, CS, or VF of participants with LP or NLP vision.

Variable	Cluster 1 (*N* = 10)	Cluster 2 (*N* = 7)	Cluster 3 (*N* = 9)	*P* value (overall)
**Raw-WPNS points**	**6.50 **±** 7.21, 2.82 (10)**	**53.16 **±** 16.30, 57.16 (7)**	**121.14 **±** 22**.**72, 114.71 (9)**	**<0** **.** **001**
**Static VA (logMAR)**	**1.77 **±** 0.43, 1.60 (10)**	**1.61 **±** 0.21, 1.57 (6)**	**1.73 **±** 0.68, 1.50 (3)**	**0** **.** **041**
Dynamic VA (logMAR)	1.90 ± 0.25, 2.00 (9)	1.82 ± 0.36, 1.60 (5)	1.30 ± 0.14, 1.30 (2)	0.091
AULCSF (logCS)	0.17 ± 0.25, 0.06 (10)	0.19 ± 0.22, 0.16 (6)	0.36 ± 0.41, 0.28 (3)	0.189
Translational MP (%)	58.0 ± 30.0, 62.7 (9)	59.3 ± 18.3, 66.6 (4)	48.3 ± 19.2, 48.3 (2)	0.744
Radial MP (%)	65.8 ± 29.8, 67.7 (9)	43.6 ± 17.2, 52.1 (4)	59.8 ± 32.8, 59.8 (2)	0.411
LS (change in logMAR)	0.01 ± 0.08, 0.01 (10)	0.00 ± 0.11, 0.01 (6)	−0.02 ± 0.05, 0.00 (3)	0.816
GLS (change in logMAR)	0.13 ± 0.17, 0.06 (10)	0.23 ± 0.39, 0.10 (6)	0.36 ± 0.54, 0.10 (3)	0.813
GLR (change in logMAR)	0.01 ± 0.13, 0.00 (10)	0.04 ± 0.16, 0.01 (6)	0.27 ± 0.45, 0.02 (3)	0.454
**VF (%)**	**66.3 **±** 24.3, 73.3 (10)**	**70.8 **±** 25.0, 68.3 (6)**	**41.7 **±** 36.8, 45.0 (3)**	**0** **.** **004**
*Age (years)*	23.6 ± 3.0, 24.5 (10)	29.0 ± 8.6, 27.0 (7)	26.2 ± 6.6, 26.0 (9)	0.340
*Age started skiing (years)*	13.6 ± 4.5, 16.0 (10)	12.3 ± 8.6, 15.0 (7)	16.0 ± 8.9, 15.0 (9)	0.439
*Age of onset of impairment (years)*	5.5 ± 4.5, 5.0 (10)	9.9 ± 12.1, 8.0 (7)	5.9 ± 7.6, 3.0 (9)	0.917
*Total lifetime hours of skiing*	5447.6 ± 4383.9, 3796.0 (10)	4440.0 ± 4378.2, 3064.0 (7)	3625.1 ± 3023.9, 2620.0 (9)	0.447
***Number of races during the validity period***	**15.2 **±** 4.7, 16.5 (10)**	**11.0 **±** 4.4, 10.0 (7)**	**9.8 **±** 5.8, 7.0 (9)**	**0** **.** **044**
Current competition class	B3 (10.0%), B2 (90.0%)	B3 (28.6%), B2 (57.1%), B1 (14.3%)	B3 (11.1%), B2 (11.1%), B1 (77.8%)	

**Figure 2:**
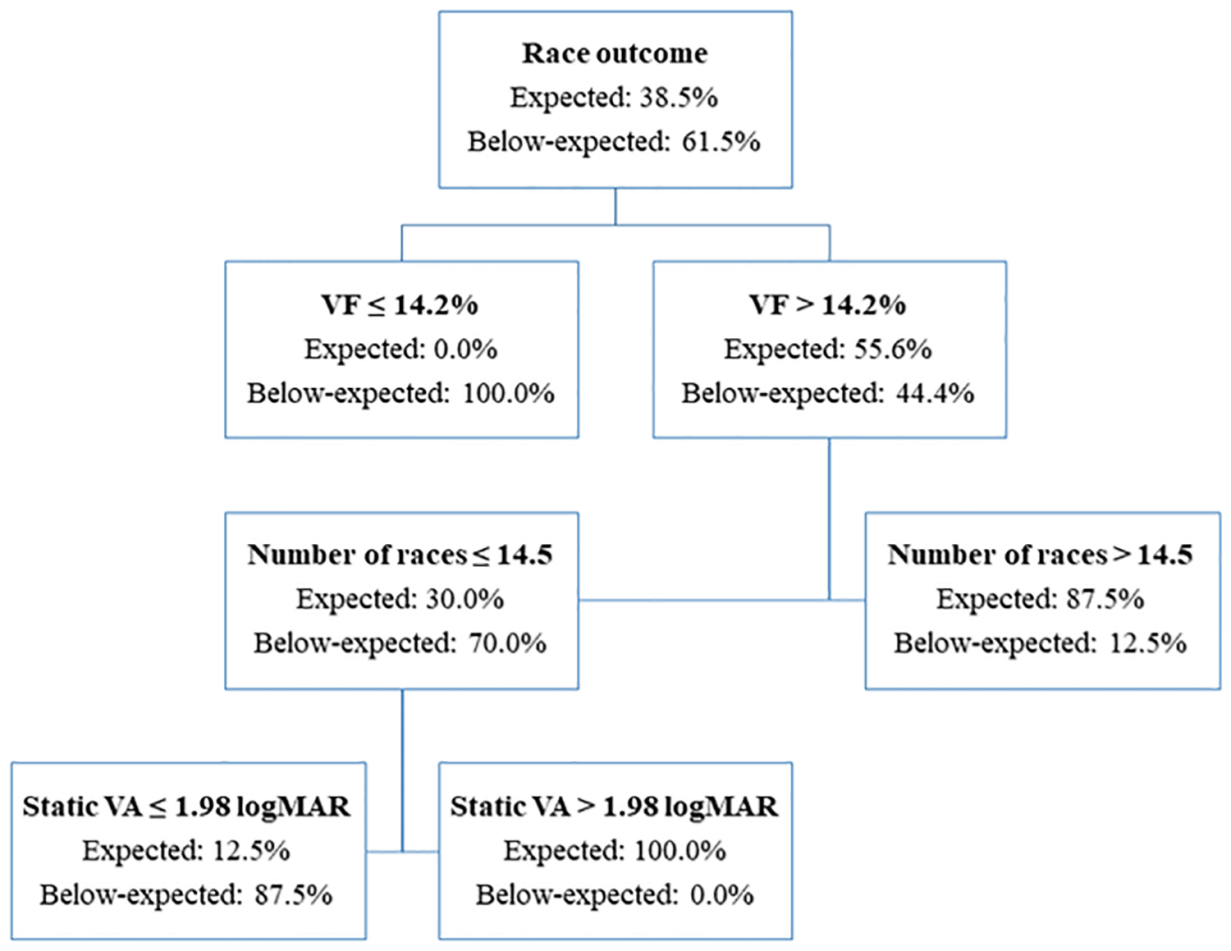
Decision tree analysis results using the C 5.0 algorithm for the Para nordic participants.

Since all the three clusters were comparable in size and were distinct in terms of average raw-WPNS points, it was possible to conduct a decision tree analysis to determine the factors that best predicted “expected” and “below-expected” performances (Figure 2). The decision tree analysis indicated that the participants who had a VF extent of ≤14.2% were more likely to perform worse compared to those with VF extent >14.2%. In participants with >14.2% VF extent, the second most significant predictor of “below-expected” performance was having competed in fewer races.

### 3.3. Para alpine skiing performance clusters

**Figure 3:**
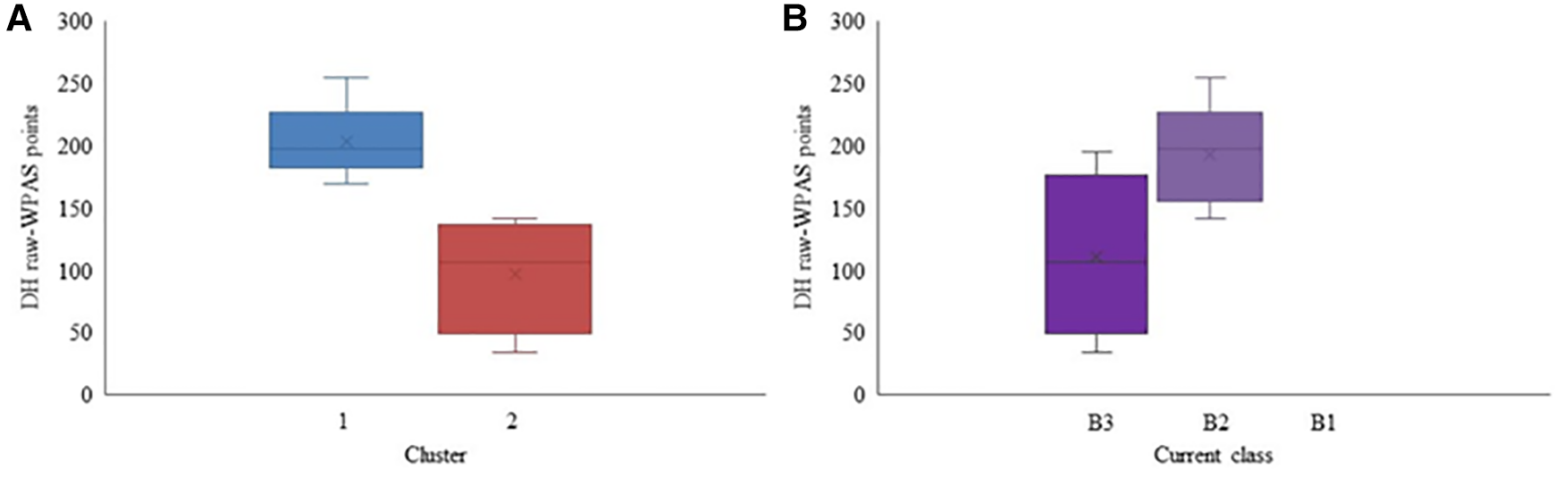
Raw-WPAS points for each cluster (**A**) and Raw-WPAS points for each current competition class (**B**) for the Para alpine DH participants. Mean value is marked with × in each box.

Hierarchical cluster analysis was conducted separately for each Para alpine discipline. Two distinct clusters were identified in the DH discipline based on the raw-WPAS DH points (*p* = 0.016) (Figure 3A), which also differed significantly in terms of the participants' dynamic VA (*p* = 0.029). Static VA, CS, and translational MP were also nearly significantly different (*p* = 0.063) between the clusters (Table 2). There were no study participants classed as B1 (>2.6 logMAR, LP or NLP VA) who competed in DH. The DH raw-WPAS points of participants based on their current classes are also included in Figure 3B for comparison purposes. There was no overall significant difference between the skiers' performance based on their current class (*p* = 0.09).

**Table 2:** Summary statistics (mean ± SD, median) of the vision and non-vision variable in Para alpine DH clusters. The values of variables that are significantly different between the clusters are bolded. The values of non-vision variables are italicized. The summary data include only actual measured values and not the arbitrarily assigned values of VA, CS, or VF of participants with LP or NLP vision.

Time	Cluster 1 (*N* = 4)	Cluster 2 (*N* = 5)	*P* value (overall)
**DH raw-WPAS points**	**97.10 **±** 46.5, 106.6 (4)**	**187.55 **±** 21.6, 195.1 (5)**	**0** **.** **016**
Static VA (logMAR)	0.65 ± 0.58, 0.67 (4)	1.38 ± 0.23, 1.40 (5)	0.063
**Dynamic VA (logMAR)**	**0.88 **±** 0.29, 0.90 (4)**	**1.80 **±** 0.33, 1.90 (4)**	**0** **.** **029**
AULCSF (logCS)	1.16 ± 0.66, 1.16 (4)	0.30 ± 0.30, 0.20 (5)	0.063
Translational MP (%)	29.6 ± 20.4, 25.7 (4)	65.0 ± 22.7, 61.0 (5)	0.063
Radial MP (%)	47.8 ± 38.0, 38.1 (4)	59.0 ± 31.6, 55.5 (5)	0.556
LS (change in logMAR)	0.07 ± 0.08, 0.04 (4)	0.07 ± 0.16, 0.02 (3)	1.000
GLS (change in logMAR)	0.09 ± 0.08, 0.08 (4)	0.15 ± 0.03, 0.14 (5)	0.413
GLR (change in logMAR)	0.05 ± 0.09, 0.01 (4)	0.07 ± 0.05, 0.06 (5)	0.286
VF (%)	50.4 ± 39.1, 48.3 (4)	57.7 ± 25.3, 55.0 (5)	0.730
*Age (years)*	32.3 ± 18.7, 26.5 (4)	27.4 ± 11.9, 20.0 (5)	1.000
*Age started skiing (years)*	15.5 ± 10.5, 11.5 (4)	18.0 ± 8.9, 17.0 (5)	0.413
*Age of onset of impairment (years)*	9.3 ± 11.6, 5.5 (4)	4.8 ± 5.6, 3.0 (5)	0.730
*Total lifetime hours of skiing*	6136.5 ± 5982.3, 5568.0 (4)	5243.2 ± 4162.5, 4320.0 (5)	1.000
*Number of races during the validity period*	7.0 ± 2.8, 8.0 (4)	6.6 ± 2.0, 6.0 (5)	0.730
Current competition class	B3 (20.0%) & B2 (80.0%)	B3 (75.0%) & B2 (25.0%)	

**Figure 4:**
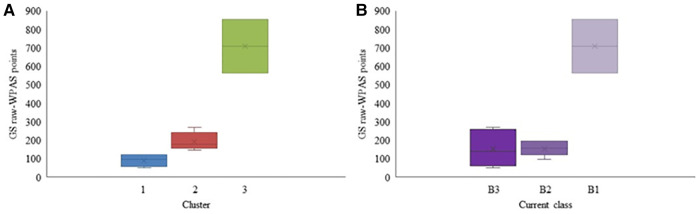
Raw-WPAS points for each cluster (**A**) and Raw-WPAS points for each current competition class (**B**) for the Para alpine GS participants. Mean value is marked with × in each box.

Three distinct groups were identified in GS that performed significantly different in terms of the GS raw-WPAS points (*p* < 0.05) (Figure 4A). The GS raw-WPAS points of participants based on their current classes are also provided for comparison purposes as Figure 4B. There was no overall significant difference among the skiers' performance based on their current class (*p* = 0.09).

Static VA and CS were the only variables found to be significantly different between the GS clusters (static VA: *p* = 0.019 and CS: *p* = 0.043). None of the other vision or non-vision variables were different between the clusters (Table 3). Post-hoc analysis showed that static VA was significantly different between clusters 2 and 3 (adjusted significance: *p* = 0.019), but there was no difference in static VA between clusters 1 and 2 (adjusted significance: *p* = 0.253). Similarly, CS was significantly different only between clusters 1 and cluster 3 (adjusted significance: *p* = 0.041). Only cluster 3 had participants with LP or NLP vision.

**Table 3:** Summary statistics (mean ± SD, median) of the vision and non-vision variables in Para alpine GS clusters. The values of variables that are significantly different among the clusters are bolded. N/M indicates that the particular variable was non-measurable. The values of non-vision variables are italicized. The summary data include only actual measured values and not the arbitrarily assigned values of VA, CS, or VF of participants with LP or NLP vision.

Variable	Cluster 1 (*N* = 5)	Cluster 2 (*N* = 8)	Cluster 3 (*N* = 2)	*P* value (overall)
**GS raw-WPAS points**	**90.34 **±** 32.70, 95.80 (5)**	**194.70 **±** 46.10, 187.17 (8)**	**563.66 & 854.02**	**0** **.** **003**
**Static VA (logMAR)**	**0.99 **±** 0.43, 1.06 (5)**	**1.34 **±** 0.53, 1.52 (8)**	**NLP and LP**	**0** **.** **019**
Dynamic VA (logMAR)	1.26 ± 0.42, 1.20 (5)	1.67 ± 0.64, 1.85 (6)	N/M	0.197
AULCSF (logCS)	0.72 ± 0.50, 0.64 (5)	0.42 ± 0.64, 0.19 (8)	N/M	0.043
Translational MP (%)	40.4 ± 14.4, 43.8 (5)	67.9 ± 36.8, 75.7 (7)	N/M	0.120
Radial MP (%)	50.2 ± 37.3, 51.5 (5)	61.6 ± 23.5, 55.5 (7)	N/M	0.416
LS (change in logMAR)	0.12 ± 0.11, 0.10 (4)	0.08 ± 0.16, 0.04 (6)	N/M	0.588
GLS (change in logMAR)	0.09 ± 0.08, 0.08 (5)	0.24 ± 0.19, 0.16 (8)	N/M	0.122
GLR (change in logMAR)	0.05 ± 0.09, 0.02 (5)	0.04 ± 0.08, 0.03 (8)	N/M	0.941
VF (%)	55.7 ± 39.0, 56.7 (5)	53.6 ± 22.8, 48.3 (8)	N/M	0.123
*Age (years)*	25.4 ± 8.8, 20.0 (5)	29.1 ± 14.6, 25.5 (8)	28.0 & 34.0	0.644
*Age started skiing (years)*	15.6 ± 9.2, 12.0 (5)	16.3 ± 8.2, 14.5 (8)	10.0 & 25.0	0.911
*Age of onset of impairment (years)*	9.0 ± 10.0, 8.0 (5)	3.1 ± 4.1, 1.0 (8)	9.0 & 0.0	0.403
*Total lifetime hours of skiing*	4842.8 ± 3873.1, 4320.0 (5)	4380.0 ± 4829.2, 1960.0 (8)	2816.0 & 1520.0	0.907
*Number of races during the validity period*	10.3 ± 3.3, 12.0 (5)	8.0 ± 3.0, 7.5 (8)	12.0 & 4.0	0.333
Current competition class	B3 (60.0%), B2 (40.0%)	B3 (37.5%), B2 (62.5%)	B1 (100.0%)	

**Figure 5:**
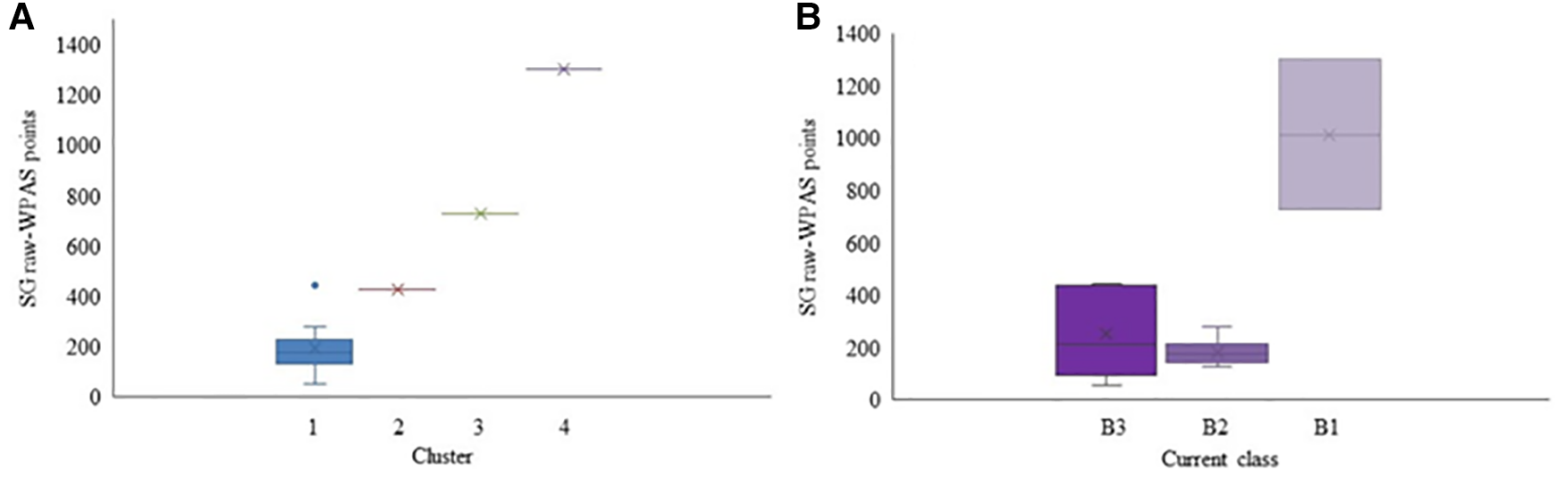
Raw-WPAS points for each cluster (**A**) and Raw-WPAS points for each current competition class (**B**) for the Para alpine SG participants. Mean value is marked with × in each box.

Based on the SG raw-WPAS points, four distinct groups were identified that performed significantly differently (*p* = 0.038) (Figure 5A). Figure 5B also includes the SG raw-WPAS points of participants based on their current classes for comparison purposes. There was no significant difference among the skiers' performance based on their current class (*p* = 0.09).

Only static VA was significantly different (*p* = 0.039) between the clusters (Table 4). Cluster 2, 3, and 4 had only one participant each. Participants in clusters 3 and 4 had NLP and LP vision, respectively (B1 class).

**Table 4:** Summary statistics (mean ± SD, median) of the vision and non-vision variables in Para alpine SG clusters. The values of variables that are significantly different among clusters are bolded. N/M indicates that the particular variable was non-measurable. The values of non-vision variables are italicized.

Variable	Cluster 1 (*N* = 10)	Cluster 2 (*N* = 1)	Cluster 3 (*N* = 1)	Cluster 4 (*N* = 1)	*P* value (overall)
**SG raw-WPAS points**	**151.60 **±** 73.70, 155.5 (10)**	**425** **.** **90**	**727.51**	**1299.42**	**0** **.** **038**
**Static VA (logMAR)**	**1.11 **±** 0.53, 1.32 (10)**	**1** **.** **64**	**NLP**	**LP**	**0** **.** **039**
Dynamic VA (logMAR)	1.40 ± 0.56, 1.40 (9)	N/M	N/M	N/M	*n/a
AULCSF (logCS)	0.66 ± 0.62, 0.54 (10)	0.01	N/M	N/M	0.206
Translational MP (%)	54.8 ± 30.8, 53.3 (10)	100.0	N/M	N/M	0.202
Radial MP (%)	56.6 ± 32.0, 55.3 (10)	54.5	N/M	N/M	0.751
LS (change in logMAR)	0.07 ± 0.10, 0.02 (7)	−0.08	N/M	N/M	0.118
GLS (change in logMAR)	0.11 ± 0.07, 0.13 (10)	0.06	N/M	N/M	0.338
GLR (change in logMAR)	0.05 ± 0.07, 0.02 (10)	−0.06	N/M	N/M	0.110
VF (%)	50.7 ± 30.8, 48.3 (10)	40.0	0.0	N/M	0.254
*Age (years)*	29.3 ± 13.6, 23.5 (10)	26.0	28.0	34.0	0.796
*Age started skiing (years)*	15.8 ± 9.2, 12.5 (10)	22.0	10.0	25.0	0.563
*Age of onset of impairment (years)*	6.1 ± 8.1, 3.0 (10)	0.0	9.0	0.0	0.614
*Total lifetime hours of skiing*	5556.2 ± 4460.7, 4560.0 (10)	2240.0	2816.0	1520.0	0.535
*Number of races during the validity period*	8.4 ± 30.0, 8.5 (10)	2.0	7.0	3.0	0.137
Current competition class	B3 (40.0%) & B2 (60.0%)	B3 (100.0%)	B1 (100.0%)	B1 (100.0%)	

* n/a indicates that statistical analyses could not be completed. The summary data include only actual measured values and not the arbitrarily assigned values of VA, CS, or VF of participants with LP or NLP vision.

**Figure 6:**
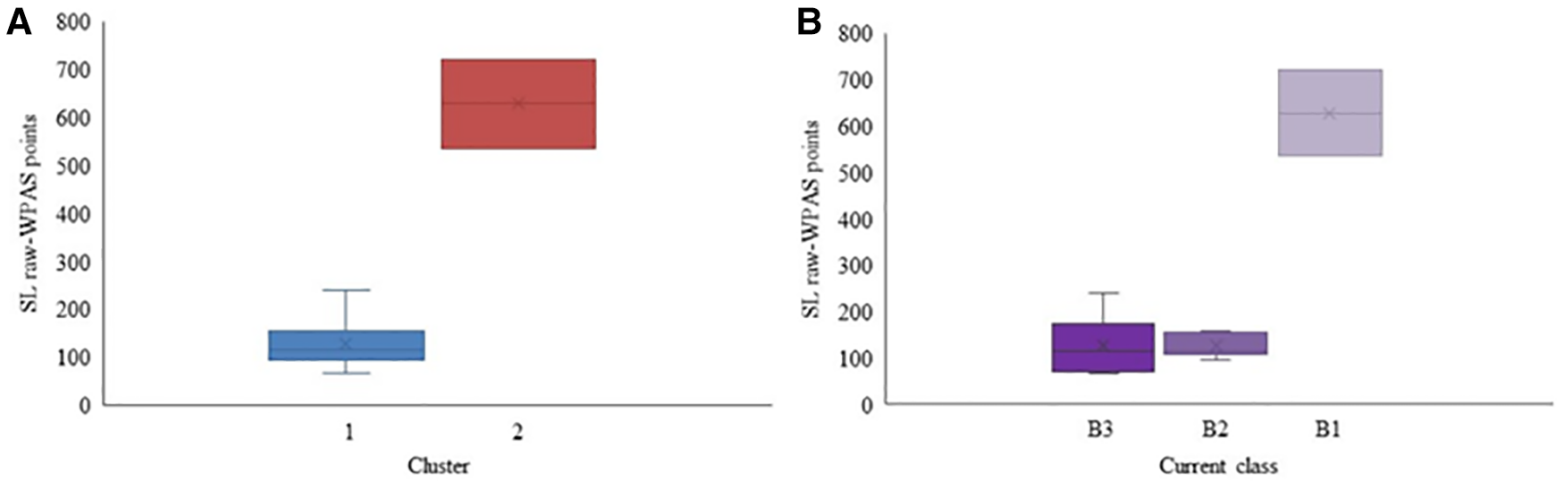
Raw-WPAS points for each cluster (**A**) and Raw-WPAS points for each current competition class (**B**) for the Para alpine SL participants. Mean value is marked with × in each box.

In SL, two distinct groups were identified that performed significantly differently (*p* = 0.019) (Figure 6A). The SL raw-WPAS points of participants based on their current classes are also provided for comparison purposes as Figure 6B. There was no overall significant difference among the skiers' performance (*p* = 0.07) based on their current class.

The performance groups differed significantly in terms of the number of races (*p* = 0.038), static VA (*p* = 0.019), CS (*p* = 0.038), and VF (*p* = 0.038) of the participants (Table 5). Cluster 2 had participants with LP or NLP vision (B1 class) only.

**Table 5:** Summary statistics (mean ± SD, median) of the vision and non-vision variables in Para alpine SL clusters. The values of variables that are significantly different between the clusters are bolded. N/M indicates that the particular variable was non-measurable. The values of non-vision variables are italicized.

Variable	Cluster 1 (*N* = 13)	Cluster 2 (*N* = 2)	*P* value (overall)
**SL raw-WPAS points**	**119.90 ** **±** ** 33.40, 129.1 (13)**	**534.74 & 722.13**	**0** **.** **019**
**Static VA (logMAR)**	**1.20 ** **±** ** 0.53, 1.40 (13)**	**NLP & LP**	**0** **.** **019**
Dynamic VA (logMAR)	1.50 ± .59, 1.40 (11)	N/M	*n/a
AULCSF (logCS)	0.55 ± 0.62, 0.40 (13)	N/M	0.038
Translational MP (%)	58.8 ± 32.2, 53.3 (12)	N/M	*n/a
Radial MP (%)	56.8 ± 29.0, 55.3 (12)	N/M	*n/a
LS (change in logMAR)	0.09 ± 0.14, 0.04 (10)	N/M	*n/a
GLS (change in logMAR)	0.18 ± 0.17, 0.14 (13)	N/M	*n/a
GLR (change in logMAR)	0.05 ± 0.07, 0.02 (13)	N/M	*n/a
VF (%)	53.5 ± 28.5, 55.0 (13)	N/M	0.038
*Age (years)*	27.9 ± 12.9, 25.0 (13)	28.0 & 34.0	0.381
*Age started skiing (years)*	16.0 ± 8.6, 13.0 (13)	10.0 & 25.0	1.000
*Age of onset of impairment (years)*	5.3 ± 7.7, 3.0 (13)	9.0 & 0.0	1.000
*Total lifetime hours of skiing*	4751.2 ± 4452.6, 2240.0 (13)	2816.0 & 1520.0	0.800
***Number of races during the validity period***	**15.6 ** **±** ** 21.0, 11.0 (13)**	**5.0 & 2.0**	**0** **.** **038**
Current competition class	B3 (46.2%) & B2 (53.8%)	B3 (100.0%)	

* n/a indicates that statistical analyses could not be completed. The summary data include only actual measured values and not the arbitrarily assigned values of VA, CS, or VF of participants with LP or NLP vision.

Decision tree analysis was not possible in the Para alpine data due to 1) non-comparable cluster sizes and 2) smaller sample sizes compared to Para nordic sample size.

## 4. Discussion

This project aimed at grouping Para nordic and Para alpine participants into clusters with highly similar skiing performances within each cluster, which at the same time were dissimilar to participants of different clusters. In Para nordic, three distinct clusters were identified based on the raw-WPNS points with comparable cluster sizes. Para nordic participants in clusters 1 and 2 had better skiing performances, better static VA, and larger VF compared to participants in cluster 3, which had most of the participants currently classified as B1. Cluster 1 participants also competed in a significantly higher number of races in the 2016–2018 season compared to cluster 3 participants. Cluster 1 and cluster 2 were not significantly different in any of the vision or non-vision aspects. The decision tree analysis also resulted in choosing the VF extent and static VA as the second and third best predictors of raw-WPNS points, respectively.

Among the Para alpine disciplines, DH is considered as a speed event, and GS, SG, and SL require progressively increasing technical skills compared to DH. The number of the Para alpine participants in each discipline also varies, with a high number of athletes participating in more technical events (*N* = 15 in SL and GS, *N* = 13 in SG) compared to DH (*N* = 9). There are also differences in the participation of skiers with most severe vision impairments among these disciplines. The Para alpine participants classified as B1 competed in all Para alpine events except the DH. Like Para nordic, the cluster analysis in Para alpine participants suggests that participants with better skiing performances also have better static VA, especially in the disciplines requiring more technical skill. SL is the most technical discipline, in which the worst-performing cluster had no measurable vision (i.e., NLP and LP visual acuity and 0.0% of VF extent). In DH, dynamic VA was significantly better in the group with better raw-WPAS points, which also had a higher number of participants with better vision overall (75% currently classified as B3).

Considering the recommended sample sizes for population-based studies, the Para nordic and Para alpine studies had smaller sample sizes (31, 32). However, during the time period when this analysis was conducted, there were only 46 and 34 registered and active Para nordic and Para alpine skiers qualified to compete for the World Championships events in the world, out of which 23.9%, 41.3%, and 34.8% of Para nordic and 5.9%, 55.9%, and 38.2% of Para alpine skiers are classified as B1, B2, and B3, respectively. These studies included 56.5% and 44.1% of the entire Para nordic and Para alpine skiers' populations, respectively. The percentages of study participants classified as B1, B2 and B3 were 30.8%, 53.9%, and 15.4% in the Para nordic and 13.3%, 46.7%, and 40.0% in the Para alpine. Therefore, considering the number and uniqueness of our population, the study samples were reasonably representative of the skiers' populations.

Although the studies had representation of participants from all three classes, one limitation of these studies is that in both the Para nordic and Para alpine studies, there was not much representation of participants with visual functions in the lower end of the B2 class and upper end of the B1 class. The Para nordic study sample had only one participant in the lower end of the B2 class static VA (i.e., static VA from 2.30 to 2.60) and one participant the upper end of the B1 class static VA (2.60 to 2.90 logMAR). No Para alpine participants who had static VA in these ranges participated in the study. Therefore, it is difficult to draw conclusions regarding the impact of the VI on skiing performance in these particular groups of skiers based on these studies. It is also interesting to note that the nordic participant in the lower static VA end of B2 was in the worst-performing cluster, yet the nordic participant with static VA in the upper end of B1 was in the best-performing cluster despite starting training at similar ages, having similar training volumes, and competing in fewer races compared to the B2 participant. Both these participants had acquired visual impairments, which were also progressive. However, the better performing B1 participant had an early onset of VI (5 years), compared to the B2 participant (11 years), which might indicate that the adaptation level of the participant to the VI could have played a role in the skiing performance.

The poorer performances (worst average raw-WPNS and raw-WPAS points) of the participants in B1 class in both Para nordic and Para alpine data were consistent with the reports of comparatively poor performances of B1 athletes in both Para judo (33) and Para swimming (34, 35), indicating that athletes with the most severe VI perform differently compared to partially sighted athletes in all these sports. The performances between B2 and B3 participants were not significantly different in either of the Para nordic or Para alpine studies, which suggests that Para nordic and Para alpine skiers with measurable vision do not differ significantly in terms of their skiing performances. The results of this study would suggest that Para nordic and Para alpine skiers with LP or NLP vision should be in one class and that the skiers with quantifiable static VA should be in a different class. Thus, there could be two distinct classes in both Para nordic and Para alpine skiing: 1) eligible participants with static VA better than 2.60 logMAR and 2) participants with no quantifiable vision (LP and NLP). As the Para nordic study had only limited participation (*N* = 2) and the Para alpine study did not have participation of skiers with static VAs ranging from 2.30 to 2.90 logMAR, follow up studies are essential to assess the performance of these new classification systems, especially in populations of skiers with static VAs in this range. Additional consultation with Para athletes and coaches of Para athletes with static VAs ranging from 2.30 to 2.90 logMAR should also be sought before any final decisions are made about the sport classes proposed here. The decision to change the sport class rules remains at the sole discretion of the Para nordic and Para alpine sport governing bodies. In addition, if new eligibility criteria are used, the final decision on the sports classes can only be made after considering both criteria, and it would not be fair to combine a new group of skiers with the group of currently eligible Para skiers as their performance levels have not been compared with the currently classified skiers. It should also be noted that these study results can only be used to provide initial recommendations based purely on the data that was collected and available to the researchers. The ultimate decision on class allocation should be made considering all the factors related to the practicality issues, including the sport technical rules, that would be involved in the implementation of classification systems in Paralympic sports. It should also be noted that rigorous follow up studies must be conducted to evaluate the performance of any new classification systems that are implemented, to determine if they are working as anticipated and to modify them if necessary.

## Data Availability

The datasets presented in this article are not readily available because of the possibility of compromising the anonymity of the participants considering the small and unique populations involved in these studies. Requests to access the datasets should be directed to, kristine.dalton@uwaterloo.ca.
